# The Prognostic Value of White-Matter Selective Double Inversion Recovery MRI Sequence in Multiple Sclerosis: An Exploratory Study

**DOI:** 10.3390/diagnostics11040686

**Published:** 2021-04-12

**Authors:** Francesco Crescenzo, Damiano Marastoni, Anna Isabella Pisani, Agnese Tamanti, Caterina Dapor, Annalisa Colombi, Alessandro Brillo, Roberta Magliozzi, Francesca Benedetta Pizzini, Marco Castellaro, Massimiliano Calabrese

**Affiliations:** 1 Department of Neurosciences, Biomedicine and Movement Sciences, University of Verona, 37134 Verona, Italy; francescocrescenzo89@gmail.com (F.C.); damianomarastoni7@gmail.com (D.M.); annaisabella.pisani@univr.it (A.I.P.); agnese.tamanti@univr.it (A.T.); caterina.dapor@univr.it (C.D.); annalisacolombi07@gmail.com (A.C.); brillo-10@hotmail.it (A.B.); roberta.magliozzi@univr.it (R.M.); marco.castellaro@gmail.com (M.C.); 2 Neurology Unit, Mater Salutis Hospital, Legnago, 37045 Verona, Italy; 3 Department of Diagnostics and Public Health, University of Verona, 37134 Verona, Italy; francescabenedetta.pizzini@univr.it

**Keywords:** multiple sclerosis, white matter, double inversion recovery, DIR, MRI

## Abstract

Using a white-matter selective double inversion recovery sequence (WM-DIR) that suppresses both grey matter (GM) and cerebrospinal fluid (CSF) signals, some white matter (WM) lesions appear surrounded by a dark rim. These dark rim lesions (DRLs) seem to be specific for multiple sclerosis (MS). They could be of great usefulness in clinical practice, proving to increase the MRI diagnostic criteria specificity. The aims of this study are the identification of DRLs on 1.5 T MRI, the exploration of the relationship between DRLs and disease course, the characterization of DRLs with respect to perilesional normal-appearing WM using magnetization transfer imaging, and the investigation of possible differences in the underlying tissue properties by assessing WM-DIR images obtained at 3.0 T MRI. DRLs are frequent in primary progressive MS (PPMS) patients. Amongst relapsing-remitting MS (RRMS) patients, DRLs are associated with a high risk of the disease worsening and secondary progressive MS (SPMS) conversion after 15 years. The mean magnetization transfer ratio (MTR) of DRLs is significantly different from the lesion without the dark rim, suggesting that DRLs correspond to more destructive lesions.

## 1. Introduction

Multiple sclerosis (MS) is a chronic immune-mediated disorder of the central nervous system (CNS), which involves both white matter (WM) and grey matter (GM), and is the primary cause of non-traumatic neurological disability among young adults [1].

It is characterized by heterogeneity in histopathological features (parenchymal lymphocytic infiltration, demyelination, meningeal inflammation, axonal loss, neurodegeneration) and clinical course (relapsing-remitting MS and secondary progressive MS) [2].

Magnetic resonance imaging (MRI) is an actual paraclinical test to support MS diagnosis and for monitoring its activity over time. Beyond focal WM acute demyelination and tardive tissue loss, the conventional MRI sequences (T2- and T1-weighted imaging) are mostly insensitive to the conspicuous diversified pathological mechanisms of MS. Therefore, non-conventional MRI techniques have emerged to estimate the disease burden more accurately and identify the predictors of long-term prognosis [3].

Double inversion recovery (DIR) is an MRI sequence that permits obtaining brain WM or GM selective imaging while simultaneously suppressing the signal deriving from one of these tissues and cerebrospinal fluid (CSF) [4]. The useful role of DIR with WM and cerebrospinal fluid (CSF) signal suppression in the MS diagnostic work-up to fulfil criteria for dissemination in space detecting cortical lesions (CLs), distinctive focal demyelinating areas of the cerebral cortex, is well known [5,6]. Moreover, it establishes the relationship between the cortical damage load highlighted by GM-selective DIR and disease disability progression [7].

An appropriate choice of inversion times permits suppressing both CSF and GM signals to enhance WM [4]. The value of this technique in MS was investigated in one single study emphasizing the presence of a hypointense signal at the border (“dark rim”) of a subset of WM MS lesions, and hence improved the specificity of diagnostic criteria [8].

No data about the relationship between the dark rim lesions (DRLs) recognised by white-matter selective double inversion recovery (WM-DIR) and disease evolution are reported to our knowledge so far.

Therefore, based on the availability of previously acquired radiological data and a long-term clinical follow-up, we proposed that a lesion surrounded by a dark rim may reflect more destructive tissue damage related to the presence of chronically activated microglia. To confirm our hypothesis, we performed an explorative study aiming to describe the occurrence of DRLs at 1.5 Tesla (T) MRI in a cohort of relapsing-remitting MS (RRMS) and primary progressive MS (PPMS) patients, and investigate the relationship between the presence of DRLs, the poor clinical outcomes and the occurrence of secondary progressive MS (SPMS) over 15 years. Finally, in a cohort of patients that underwent a 3T MRI scan, we aimed to provide quantitative estimates of microscopic tissue damage of DRLs using magnetization transfer-based imaging (for the hypothesis that the degree of demyelination and axonal loss is greater in lesions with a dark rim than in those without a dark rim).

## 2. Materials and Methods

### 2.1. Study Population

Two different study populations were included in the study (Table 1).

**A.**  A total of 107 MS (89 RRMS, 18 PPMS) patients were monitored at the MS Specialist Centre of the University Hospital of Verona, Italy and, having at least 15 years of follow-up since their first MRI examination (performed between 2004 and 2005), were involved in the study. The inclusion criteria were a diagnosis of MS [9], the availability of a 1.5T MRI including T1-weighted-MPRAGE (T1w), fluid attenuated inversion recovery (FLAIR) and WM-DIR sequences; 15 years of clinical follow-up including neurological examination using the expanded disability status scale (EDSS) assessment at least once every year [10]. RRMS patients were considered retrospectively to have entered the SP phase when their EDSS increased by at least 1.5 points from baseline EDSS 0, 1 point from EDSS 1.0–5.0, and 0.5 points from EDSS 5.5, independently of clinical relapses over a 6–12 month interval [11]. These patients were grouped into “DRL+” and “DRL−” based on the presence and absence of DRLs, respectively. Demographical, clinical, and MRI characteristics containing data on cortical damage in each of these groups are summarized in Table 2.

At the time of image acquisition, 49 patients were receiving medical treatment with interferon beta, 33 with glatiramer acetate, 14 with azathioprine, and 3 with mitoxantrone.

**B.**  A total of 40 RRMS patients (Table 1) according to the most recent diagnostic criteria [6] underwent a 3.0 T MRI scan between 2019 and 2020 including, in addition to WM-DIR, magnetization transfer-based imaging to identify in-vivo pathological differences between lesions with a dark rim and those without a dark rim. None of them had objective signs of disease activity in the past six months. They were treated with dimethyl fumarate (21 patients), fingolimod (9 patients), natalizumab (1 patient), and ocrelizumab (1 patient).

### 2.2. MR Imaging Acquisition

1. White-matter selective DIR, FLAIR and T1w sequences were obtained using PAchieva scanners (, Philips Healthcare, Best, The Netherlands) at both MRI field strengths. For the sake of clarity, the WM-DIR sequence used in this work serves the same purpose as the GM-DIR sequence used in [8]. The DIR sequence is usually used for cortical lesion load assessment that involves mainly the GM tissue, whereas the WM-DIR sequence enhances the WM tissue and permits the detection of DRLs. Therefore, we will refer to WM-DIR in the forthcoming paragraphs.
  (a) At 1.5 T MRI scan, a white-matter selective DIR was acquired with 2D contiguous axial slices with repetition time (TR) 22,000 ms, echo time (TE) 25.0 ms, inversion tim1 (TI1) 2.542 ms, inversion time-2-delay (TI2) 605 ms, voxel size 0.9 × 0.9 mm, slice thickness 3 mm, recon matrix 256 × 256, acquisition matrix 178 × 178 mm 3D FLAIR sequences with TR 10,000 ms, TE 120 ms; TI 2500 ms, voxel size 0.9 × 0.9 mm, slice thickness 3 mm, recon matrix 256 × 256;T1wimages were acquired with TR 25 ms, TE 5 ms, voxel size 0.9 × 0.9 mm, and slice thickness 2.4 mm.
  (b) At 3.0 T MRI scan, the acquisition protocol consisted of 3D images with a voxel size of 1 mm isotropic with TR/TE 5500/312 ms, TI1/TI2 3060/815 ms and matrix 240 × 240; 3D FLAIR with a voxel size of 1 mm isotropic TR/TE 8000/292 ms, TI 2350 ms and matrix 240 × 240; 3D T1w Mwith TR 8.4 ms, TE 3.7 ms and matrix 240 × 240.
2. Magnetization transfer imaging (MTI) (3D FFE dual-echo sequence with a voxel size of 1.5 mm, TR6.4 ms, TE1/TE22.7/4.2 ms, FA 9°, with and without magnetization transfer pulses of angle 360°, duration 16 ms and offset frequency 1 kHz) at 3.0 T was acquired. The MTI acquisitions were used to compute magnetization transfer ratio (MTR) maps. First, the two echoes were averaged to improve the signal to noise ratio. MTR maps were computed as by Liu et al. [12].

### 2.3. MRI Analysis

**Figure 1:**
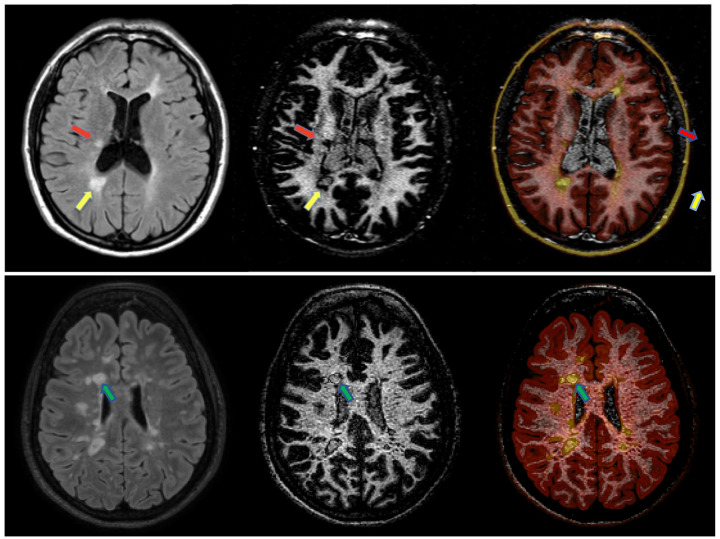
The first row describes the visualization, at 1.5 T, of a typical MS WM lesion on fluid attenuated inversion recovery (FLAIR) (**left**), characteristically surrounded by a dark rim (DRL) on a white-matter selective double inversion recovery sequence (WM-DIR) (middle, yellow arrow); a uniform hypointensity on WM-DIR (no-DRL) characterizing another WM lesion can be observed (red arrow). On the right an example of a color-coded FLAIR–WM-DIR fusion image to show where the “dark rim” is exactly located with respect to the FLAIR-visible contours. The second row shows a 3T example of DRL (green arrow) on WM-DIR (middle), FLAIR (left), and FLAIR-WM-DIR fusion (**right**).

(a) On 1.5 T MRI scans, each supratentorial WM lesion was first identified on FLAIR and then analyzed for the presence of a dark rim on the WM-DIR using medical images processing, analysis and visualization (MIPAV) v. 7.0.1 Dark rim lesions (DRLs) were defined as having a complete dark rim around the lesion on the WM-DIR sequence, and internal isointensity to extralesional WM, visible on consecutive slices (Figure 1).

The cortical thickness was calculated using FreeSurfer, a software based on a T1-weighted image (using a semi-automatic procedure with lesions filling to correct topological defects in the cortical surface due to juxtacortical lesions. Cortical lesions were evaluated on conventional DIR sequences following the consensus of recommendations [13].

(b) 3.0 T MRI scans underwent N4 bias field correction [14], and were registered to the WM-DIR images space, using the anatomical T1-weighted space as an intermediate step with the affine registration function of the ANTs toolbox [15].

At least one DRL and one lesion without a dark rim (no-DRL) were selected for each individual, to control intra-patient variability. DRLs cores and no-DRLs were segmented on 3.0 T WM-DIR, using manual and semi-automatic segmentation with an ITK-SNAP tool [16].

**Figure 2:**
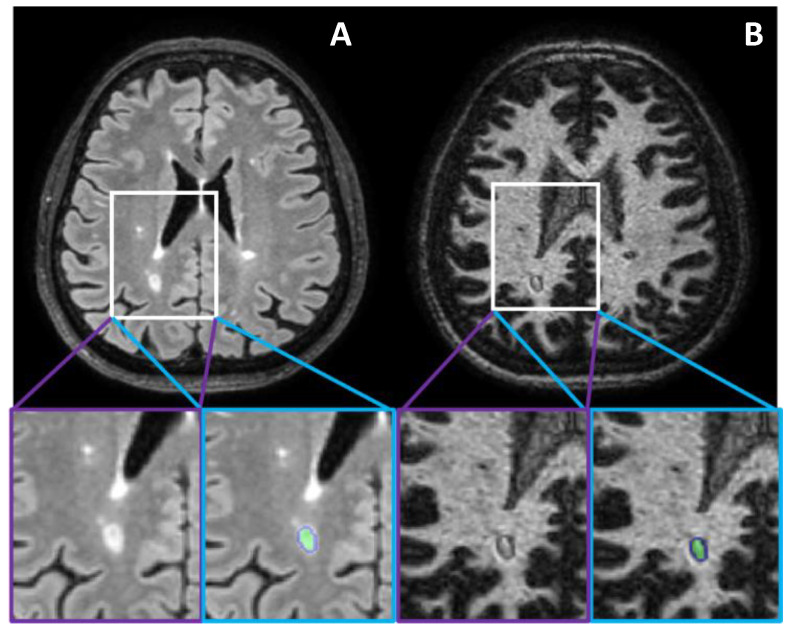
Illustrative FLAIR (**A**) and WM-DIR (**B**) images at 3.0 T of segmentation of the core of DRL with a 2-pixel-disk dilatated element to cover the dark rim.

Each segmented lesion’s edge was then dilated with a 2D disk element of 2 pixels radius, with an in-house written code implemented in MATLAB ver. R2018b. For DRLs, this dilated edge covered the characteristic dark rim surrounding the lesion core (Figure 2).

At the same time, in the case of no-DRLs, it identified an edge region of normal-appearing WM (NAWM) encircling the no-DRL on the WM-DIR image. The segmentations of lesions and boundary tissues (dark rim and edge of NAWM for DRL, and no-DRL, respectively) were used to retrieve the average MTR values in the maps’ corresponding voxels.

All MRI scans were viewed and processed by a trained rater (F.C.), continuously supervised by a neurologist with extensive experience in neuroimaging (M.C. (Massimiliano Calabrese)).

### 2.4. Statistical Analysis

The Kolmogorov test was used to test for the normal sample distributions. The Mann–Whitney–Wilcoxon test was used to compare populations for their lesion number, lesion volume, EDSS at baseline (T0), and EDSS change over 15 years (T15). The Kruskal–Wallis test was used to compare more than two sample data. The Dunn post-hoc test was used to account for multiple comparisons.

Pearson’s chi-squared test was applied to identify the difference between qualitative variables. The pairwise univariate Spearman’s rank correlation index was used to evaluate the relationship between the DRL number and other variables.

The DRL number percentage (%DRL-number) was categorized using the own quartile distribution. Therefore, patients were sorted by %DRL-number and then stratified into three categories: patients with a %DRL-number equal to the first quartile and greater than the third quartile were included in the first (0% DRL-number; 44 patients) and third categories, respectively (>33% DRL-number; 20 patients), while the remaining ones were included in the second category (0% < DRL-number ≤ 33%; 43 patients). The same approach was applied to categorized %DRL-volume in three categories. Multivariable logistic regression analysis, with backward stepwise model selection, was used to estimate the association between demographic, clinical and radiological parameters at T0, with the disability accumulation at T15.

Age at onset, gender, EDSS, CLs number, global cortical thickness, the total WM lesion number (or total WM lesion volume) at T0, and the %DRL-number (or %DRL-volume) at T0 categories were treated as independent variables, with their effect, which was expressed by the odds ratio, on the outcome of “significant” EDSS change. Considering the long follow-up period, the EDSS change was considered “significant” if its increase at T15 was at least 2 points. This value was chosen to obtain a balanced distribution of the study population (57 patients with EDSS change < 2 vs. 50 patients with EDSS change ≥ 2). A multivariable Cox regression (stepwise approach) was used to investigate the risk of developing SPMS over 15 years in RRMS patients with DRLs.

Proportional hazard assumption was checked by statistical tests [17]. Statistical analyses were performed using R). A value of *p* < 0.05 was considered statistically significant.

## 3. Results

No differences between Group DRL+ and Group DRL- were observed at T0 in gender distribution and age.

### 3.1. Dark Rim Lesion(S) (DRL) Assessment and Association with Clinical and MRI Variables at Baseline (T0)

**Figure 3:**
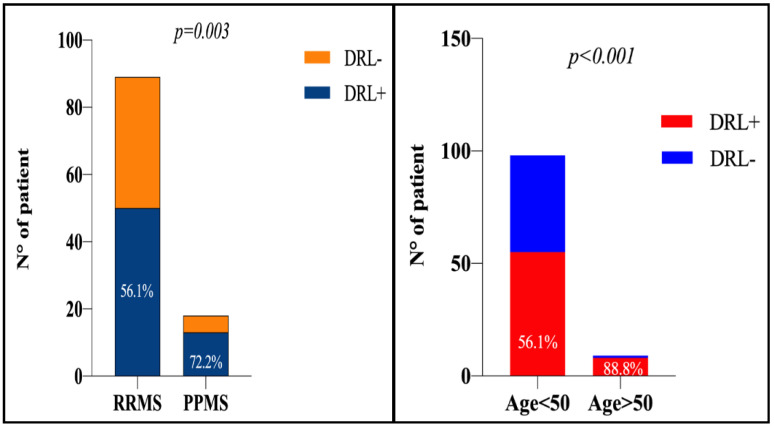
Histogram of the dark rim lesion’s distribution according to MS phenotype (**left**) and age of the patient (**right**). DRL = dark rim lesions; MS = multiple sclerosis; RRMS = relapsing-remitting MS; SPMS = secondary progressive MS.

A total number of 1260 white matter (WM) lesions were reviewed in 107 MS patients. Of these, 216 DRLs (17.2%) were identified in the 1.5-tesla WM-DIR sequence. Overall, 63/107 patients (58.9%) had at least 1 DRL. The occurrence of DRLs was 1.3-fold higher in PPMS patients (13/18, (72.2%)) than in RRMS patients (50/89 (56.1%); *p* = 0.003) with a significant difference in terms of DRL number between PP- (median 5.5, range 0–10) and RRMS patients (median 1, range 1–8; *p* = 0.002) (Figure 2). DRLs were also more frequent (8/9 (88.8%)) in older patients (age > 50) than younger (55/98 (56.1%); *p* < 0.001) (Figure 3) and their number were correlated with disease duration (rho0.41, CI 95% 0.24–0.55; *p* < 0.001).

There was also a difference in median DRL volume between PPMS and RRMS patients, with a more significant size in the latter (median 0.35, range 0.01–3.61 cm^3^) compared to the former group (0.22, range 0.01–3.13 cm^3^; *p* = 0.03).

Overall median %DRL-volume accounted for about 30% (range 5–82%) of the total WM lesion volume; it was higher (*p* = 0.064) in PPMS patients (median 35%, range 25–56%) compared to RRMS patients (median 22%; range: 5–82%), but not significantly.

### 3.2. Association between Dark Rim Lesion(s) and Clinical Outcomes after 15 Years (T15)

The EDSS change was significantly higher in Group DRL+ (median 2.25, range: 1.5–5.0) compared to Group DRL- (median 0.5, range: 0–3.5; *p* < 0.001).

According to the disease phenotype, EDSS change was also greater in RRMS patients with DRLs (median 2.5; range 0–5.5) compared to RRMS without DRLs (median 0; range 0–3.0; *p* < 0.001); and in PPMS with DRLs (median 3.5; range 1.5–6.0) compared to those without DRLs (median 3.0, range 2.0–3.5), although in the last case the result did not reach statistical significance (*p* = 0.25).

The logistic regression analysis, including clinical and MRI parameters at baseline, showed that having more than one third of WM lesions with a dark rim is an independent predictor of a “significant” EDSS change (EDSS change ≥ 2 points) at T15 (Table 3).

During the study period, 28 (31%) out of 89 RRMS converted to the SPMS.

RRMS patients with DRLs at MRI showed a higher tendency to evolve to a secondary progressive stage of the disease; at the basal MRI, 26 of them showed DRLs (92.8%), while only two did not (7.1%; *p <* 0.001).

**Figure 4:**
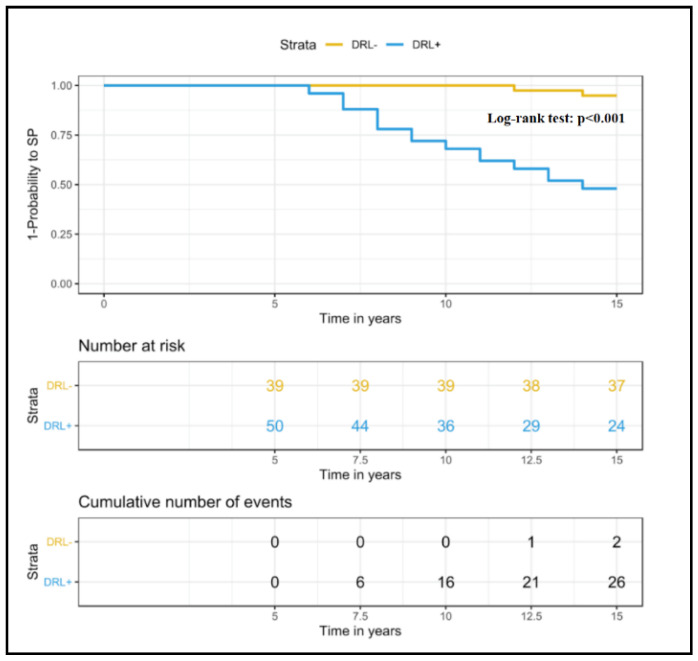
Survival curves plotted using the Kaplan–Meier method show the time from assignment to GroupDRL+ at T0 to the onset of SPMS, over a 15-year follow-up. DRL = dark rim lesions; SP = secondary progressive MS.

The survival analysis of predictive factors of developing SPMS showed a contribution of DRLs (HR 2.68, CI = 1.13–22.3; *p* = 0.001) in addition to age at disease onset, baseline EDSS and GM damage load (Table 4). Kaplan–Meier estimates showed a higher SPMS conversion at T15 among patients who presented DRL(s), compared to patients who do not have it at T0 (Figure 4).

### 3.3. 3.0. T MRI-Derived Tissue Features by Magnetization Transfer Ratio

**Figure 5:**
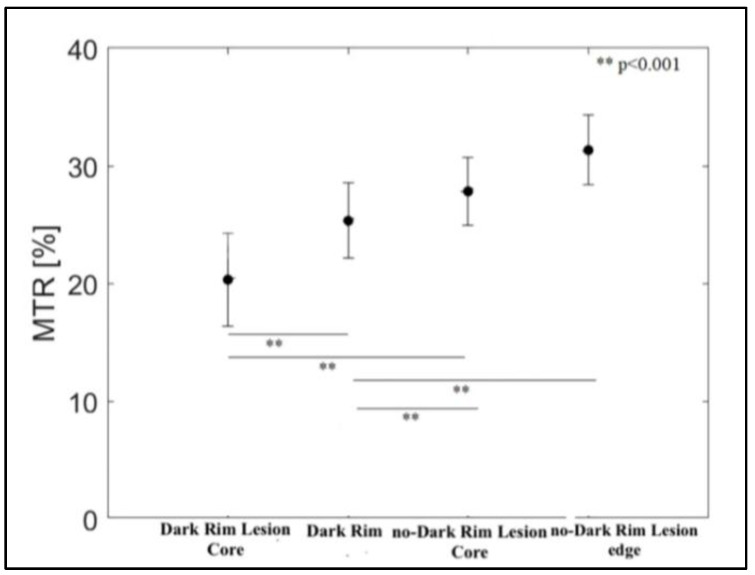
Comparison of mean MTR between DRLs (both core and rim), no-DRL core and edge, showing different MTR values across all the sampled data. MTR = magnetization transfer ratio; DRLs = dark rim lesions.

Overall, 40 discrete supratentorial DRLs and 42 comparable no-DRLs were segmented on a 3.0 T scan in the 40 RRMS patients. The following characteristics were obtained at core and rim/edge level of lesions with and without a dark rim: dark rim lesion core (mean MTR (SD), 20.31 (3.96)) and no-dark rim lesion core (27.81 (2.88), *p <* 0.001); dark rim l (25.30 (3.19) and no-dark rim lesion edge (31.33 (2.96) *p* < 0.001) (Figure 5).

## 4. Discussion

In this retrospective longitudinal study, using a WM-DIR sequence which subtracts at the same time both CSF and GM tissue, we were able to identify, in RRMS and PPMS patients, several WM lesions surrounded by a dark rim undetectable by conventional T2-weighted sequences, in 1.5 T MRI scan.

The so-called “dark rim lesions” (DRLs) were noted in more than half of the patients (59%), with higher occurrence in PPMS and older patients (>50 years), accounting, overall, for about 20% of the total WM lesions. These data are slightly different from a previous study [8] in which a similar sequence was used, and in which DRLs accounted for 35% of the total WM lesions, and almost all patients (97%) showed at least one DRL. These discordant results could be explained, at least partially, by the higher spatial resolution offered by 3.0 T with respect to 1.5 T MRI. Furthermore, the dark rim appears much thicker from our images dataset at 1.5 T, compared to those shown by Tillema et al. (due to the initial interpolation applied directly at the time of the image reconstruction in the scanner) making it difficult to identify around the small lesions, so that these appeared homogeneously dark, and consequently classified as no-DRL. Moreover, our data showing that DRLs are more frequent in progressive patients are worth noting. The evidence that some WM lesions can be rimmed both in RR- and PPMS patients came from an elegant study by Kaunzner et al., in which, matching quantitative susceptibility mapping imaging data with immunohistochemistry in ex-vivo MS brain samples, it was clearly shown that the iron within microglia and macrophages at the edge of slowly expanding lesions is the main source of rim appearance [18].

In addition to the presence of DRLs in both RRMS and PPMS patients, we considered the relative volume of DRLs, showing that, in PPMS patients, DRLs represented up to 35% of the total WM lesion volume. The not significant difference compared to the relative volume of DRLs between RRMS and PPMS groups may be influenced by the low number of progressive patients in our study.

The analysis of the 15-year follow-up revealed that both RRMS and PPMS patients with DRL at baseline experienced a worse disease evolution compared to patients without DRL; the absence of difference in EDSS changes between PPMS patients with and without DRL, respectively, could be explained by the low sample size of the PPMS cohort. Hence, a larger cohort of PPMS patients is needed to confirm the clinical results even in this group of patients.

However, the prognostic role of DRLs at baseline was emphasized by the regression model analysis. In addition to well-known prognostic variables (i.e., age, EDSS, and cortical damage), the DRLs presence and number were associated with the disability worsening, and evolution towards the disease’s secondary progressive phase.

The logistic regression analysis using the DRLs numbers as a predictor showed that a higher number of DRLs with respect to WM lesion numbers is associated with physical disability progression over 15 years. These data seem to be in line with previous pathological observations that WM lesions in MS are highly heterogeneous, and that some subtypes may play a more relevant role in the progression of the disease [19].

Although this study highlighted the possible clinical relevance of DRLs, the dark rim’s pathological nature is still unknown. For this reason, we decided to evaluate the DRLs in 40 MS patients with both a WM-DIR and MTR performed at a 3.0 T MRI scan. The MTR of both the core and the DRL rim was significantly different from the core and the WM surrounding the no-DRL core. It could be that these lesions are characterized by a subtle and evolutive degenerative process that tends to involve the core of the lesion and to spread to the surrounding WM.

Inversion recovery sequences and MTR are influenced by T1-relaxation time, which can be due to different pathological mechanisms such as demyelination, axonal loss, edema, widening of the extracellular space, glial proliferation or metals accumulation [20].

As suggested by Tillema et al. [8], the dark rim on the inversion recovery sequence could be a consequence of different T1-relaxation time between some MS lesions with long T1 times (likely because of axonal loss or edema) and adjacent NAWM, known as “boundary effects” [21].

However, other intriguing hypotheses can be advanced. Ogg and Steen stated that T1-relaxation time might be influenced by iron, as well as several macromolecules; the authors assumed that regional iron relaxivity values could reflect local differences in the magnetic and biochemical state of brain-iron and the interaction of this with tissue water [22]. This hypothesis is supported by the detection of iron accumulation in microglia at the lesion edge of chronic active lesions [23], and its reduction in oligodendrocyte of perilesional NAWM [24]. Albeit in the absence of perivascular immune infiltrates, the persistent microglial activation at the edge of these lesions releases pro-inflammatory mediators, having a fundamental role in continuous axonal damage and tissue destruction behind an intact blood-brain barrier, playing a key role in the transition from relapsing-remitting MS to the progressive phase [25]. The worse disease evolution of patients with DRLs and the higher occurrence of DRLs in progressive patients seem to support this hypothesis.

In accordance with our results, Absinta et al. have shown that chronic active lesions, in vivo, also had longer T1 times (therefore a lower MTR value) at the lesion core due to tissue destruction [26]. Furthermore, an important study by Moll et al., using combined postmortem pathology and MRI, reported that subtle reductions in MTR in perilesional WM volumes are associated with increased activated microglia density [27], as confirmed also by Colasanti et al., who combined PET with a specific radioligand for activated microglia and MRI to measure relative binding in the lesion, perilesional tissue, and surrounding NAWM of MS patients [28]. This would be in line with susceptibility-based MRI studies which demonstrated that the persistence of microglia with a pro-inflammatory activation status at the WM lesion edge represents a negative prognostic factor both in early lesion evolution and in disease progression, related to a failure in remyelination and to ongoing and diffuse tissue damage [26].

Moreover, considering that not all WM lesions showed the dark rim on the WM-DIR sequence, it can be speculated that iron-enriched microglia surrounding WM lesions might also be one of the primary sources of the dark rim; however, further studies are needed to confirm such a hypothesis. Nevertheless, to further test this hypothesis, a study comparing WM-DIR and susceptibility-based imaging is currently ongoing at our Centre.

We are aware that our study is not free from limitations. Firstly, we did not assess the potential presence of DRLs in a control group, thus preventing determining its diagnostic value. Secondly, only supratentorial lesions are considered, thus limiting a more accurate assessment of the lesion load. Thirdly, the lack of MRI scans, including a WM-DIR sequence both at first symptom onset and at the end of clinical follow-up in all patients, does not allow us to evaluate the evolution of DRLs over a long period. Fourthly, the long-term clinical outcome analysis is potentially limited by the lack of data on the effects of different treatments on the relapse rate over time in patients with and without DRLs, and both spinal cord lesional load and T1-black hole lesion volume at baseline. It is not possible to know of the possible effects of different treatments on the persistence of DRLs, although it can be presumed to not be relevant, since it is demonstrated that the efficacy of current disease-modifying treatment (DMT) on the impact of chronic inflammation cells is limited [24]. Moreover, a study conducted by Giorgio et al. demonstrated the strong association between black holes and EDSS worsening over 10 years [29], but in contrast, Filippi et al., including in the analysis MRI measures of GM damage, showed that baseline black hole lesion load does not predict worsening disability at the end of 13 years of follow-up. [30]. Further longitudinal studies are needed to evaluate the possible additional independent contribution from DRLs at baseline with respect to the worsening of disability.

Finally, MTR values are influenced by water content (i.e., edema, inflammation) and scanner parameters [31], therefore no conclusion can be drawn interpreting the data obtained on the 3.0 T to comprehend the images at 1.5 T MRI.

## Figures and Tables

**Table 1:** Demographic and clinical characteristics of populations at the time of MRI.

	MS Population 1.5 T MRI	MS Population 3.0 T MRI
Patients, number	107	40
Disease phenotype	89 RRMS	40 RRMS
	18 PPMS	
Female, number (%)	74 (69%)	21 (53%)
Age, mean (SD; range)	33.9 (10.8; 17–65)	37.8 (5.4; 21–56)
Disease duration, years to first symptom (SD; range)	3.9 (2.3; 0–9)	7.3 (6.4; 1–17)
EDSS, median (range)	1.5 (0–6)	2.0 (0–6.5)

EDSS = Expanded Disability Status Scale; MRI = magnetic resonance imaging; MS = multiple sclerosis; PPMS = primary progressive MS; RRMS = relapsing-remitting MS; SD = standard deviation.

**Table 2:** Demographical, clinical and MRI characteristics of MS patients with and without DRL at 1.5 T.

	Group DRL+	Group DRL−	*p*-Value
Patients, number (%)	63 (59%)	44 (41%)	/
Female, number (%)	45 (71%)	29 (66%)	*p* = 0.67
Age, mean (SD; range)	34.9 (11.6; 18–62)	32.5 (9.4; 18–65)	*p* = 0.25
Disease duration, mean (SD)	4.7 (2.2)	2.9 (2.1)	*p* < 0.001
EDSS, median (range)	3.0 (1–6)	1.5 (0–5.5)	*p* < 0.001
WM lesion number, median (range)	15 (2–35)	6 (1–25)	*p* < 0.001
DRL number, median (range)	3 (1–10)	/	*/*
Percentage of DRL number, median (range)	0.28 (0.4–0.8)	/	*/*
WM lesion volume (cm^3^), median (range)	4.5 (0.3–16.5)	1.0 (0.1–15.0)	*p* < 0.001
DRL volume (cm^3^), median (range)	0.9 (0.1–3.6)	/	*/*
Percentage of DRL volume, median (range)	0.3 (0.5–0.8)	/	*/*
Cortical lesions, median (range)	1.0 (1–8)	0 (0–1)	*p* = 0.75
Cortical thickness (cm^3^), mean (SD)	2.3 (0.4)	2.5 (0.2)	*p* = 0.27

DRL = dark rim lesions; EDSS = expanded disability status scale; MRI = magnetic resonance imaging; MS = multiple sclerosis; SD = standard deviation; WM = white matter.

**Table 3:** Baseline clinical and MRI variables associated with a “significant” EDSS change after 15 years.

Risk Factor	Odds Ratio	95% Confidence Interval	*p*-Value
EDSS	1.55	1.09–2.45	*p* = 0.04
CLs number	1.61	1.13–2.10	*p =* 0.004
Cortical thickness	0.11	0.01–0.86	*p* = 0.03
%DRL-number > 33%	1.32	1.03–9.21	*p* = 0.01

CLs = cortical lesions; DRL = dark rim lesions; EDSS = expanded disability status scale; MRI = magnetic resonance imaging.

**Table 4:** Hazard ratios for the development of SPMS after 15 years.

Variables	Hazard Ratio	95% Confidence Interval	*p*-Value
Age	2.91	1.74–4.21	*p* < 0.001
EDSS	1.73	1.11–2.22	*p* = 0.003
CLs number	1.25	1.01–1.57	*p* = 0.02
Cortical thickness	0.44	0.38–0.89	*p =* 0.04
Presence of DRL	2.68	1.13–22.28	*p* = 0.001

CLs = cortical lesions; DRL = dark rim lesions; EDSS = expanded disability status scale; SPMS = secondary progressive multiple sclerosis.

## Data Availability

The data supporting the findings of this study are available from the corresponding author on reasonable request.
